# An Experimental Study on Micro-Shear Clinching of Metal Foils by Laser Shock

**DOI:** 10.3390/ma12091422

**Published:** 2019-05-01

**Authors:** Xinding Li, Xiao Wang, Zongbao Shen, Youjuan Ma, Huixia Liu

**Affiliations:** School of Mechanical Engineering, Jiangsu University, Zhenjiang 212013, China; perrylxd@163.com (X.L.); szb@ujs.edu.cn (Z.S.); myj@ujs.edu.cn (Y.M.); lhx@ujs.edu.cn (H.L.)

**Keywords:** failure mode, laser shock, metal foils, micro clinching, process window

## Abstract

This work proposes a micro-shear clinching process by laser shock for joining similar and dissimilar metal foils. The joint appearance and cross-section were investigated to determine basic process parameters. The soft punch thickness was 100 μm. The numbers of laser pulses on the upper and lower foil sides were set as two and one, respectively. Joint deformation was divided into three stages and we investigated the deformation law of the joints. The process windows of the Al foil combinations were acquired to determine a reasonable range of process parameters for obtaining qualified joints. The mechanical properties and failure modes of different joints were analyzed to identify the process characteristics. Mechanical properties were related to shear test directions and were influenced by upper and lower foil thicknesses. One failure mode was observed in the parallel shear test, and four failure modes were observed in the perpendicular shear test. These modes were determined by the differences between upper and lower foil thicknesses. Results showed that the proposed process can be used to join Al and Cu foils successfully. The laws governing the mechanical properties and failure modes of dissimilar materials were similar to those governing the mechanical properties and failure modes of similar materials.

## 1. Introduction

The traditional mechanical clinching process has notable benefits, such as rapidity, wide applicability, high dependability, energy savings, and environmental friendliness. Abundant literature has been published for mechanical clinching to improve the feasibility and application of the process involving numerical and experimental investigations. Lee et al. [1] investigated the joinability of high-strength steel (H320LA) with the Al5052 alloy by finite element analysis. It was concluded that the joinability was mainly affected by the concave angle of the die, the depth of the die, and the shape of the die groove, and the strength of the joint was determined by the width of the interlock and neck thickness. Mucha et al. [2] studied the influence of the type and the thickness layout of the car-body sheets on the joint strength and found that the sheet thickness arrangement in relation to die is a very important parameter for the load-carrying ability of the joint. The author [3] further researched the influence of the process parameters on the joinability of advanced high-strength steel (H320LA) by numerical simulation, and it was determined that the groove width of the die had the greatest influence on the material flow and energy absorption during the forming process. Abe et al. [4] analyzed mechanical clinching of high-strength steel and aluminum alloy with dies for control of metal flow by numerical simulation. The results showed that the optimization of the depth of the die or the elimination of the groove of the die can avoid cracking of the high-strength steel, when high-strength steel is, respectively, used as the upper or the lower sheet. Lambiase and Ilio [5,6,7] investigated five geometric process parameters of mechanical clinching using the extensible die by numerical simulation, and built the relevant process windows; the material flow studies showed that the width of the interlock increased with the decrease of the diameter of the extensible die, and the width of the interlock was negatively correlated with neck thickness in the circumferential direction. Then based on the L27 Taguchi orthogonal design for the five geometric parameters, the interlock and joint strength were further analyzed by artificial neural network method (ANN), and the optimized process parameters were obtained by genetic algorithm (GA). Abe et al. [8] successfully achieved the mechanical clinching of two kinds of ultra-high strength steels by optimizing the die diameter and depth. Coppieters et al. [9] produced a device for testing mechanical clinching joints’ strength according to different shear and tensile ratios to analyze the actual load conditions during application. Israel [10] explored the analytical and numerical methods for mechanical clinching of thick metal sheets and found that it can approximately determine the maximum joining force of the joint, but cannot replicate the force change over stroke involved with clinching. Kaščák et al. [11] studied the mechanical clinching of dual-phase steels (DP600) by numerical simulation and analyzed the wear of the punch and the die with CrN coating. The results showed that the wear of the punch was significantly larger than that of the die. Then the author [12] studied the mechanical clinching of galvanized high-strength steel (H220PD+Z) and investigated the wear of the punch and the die with different coatings. Based on numerical simulation, Eshtayeh et al. [13] carried out the multi-objective optimization of the interlock, neck, and bottom thickness of the joint using the grey-based Taguchi method, and obtained three pairs of optimal levels with homologous confirmation. Wang et al. [14] also conducted a comprehensive analysis of the geometric parameters and process parameters of mechanical clinching and determined the eight parameters that had the greatest influence on the interlock width, neck thickness, and tensile force of the joint through a sensitivity analysis. Then through response surface methodology and a multi-objective genetic algorithm (NSGA-II), the authors conducted the multi-objective optimization with constraints and obtained the optimized process parameters and joints. Zhang et al. [15] investigated the failure mechanism of the joints. The results showed that the fracture regions are always concentrated in the indentations of the lower sheets, and the ratio of the neck fretting wear mode and indentation-surrounding fretting wear mode is influenced by the load level.

However, the above studies were mainly for joints produced by round tools without shearing actions. Rectangular shear clinching joining is also one of the most important mechanical clinching processes and confers shearing behaviors to workpieces. Hahn and Klemens [16] introduced the traditional multistage shear clinching process. In this process, first, two layers are stacked and fixed between a bracket and shear die. Second, shear punch cuts are made in the two layers along two parallel edges using the shear die. Third, the sheared part of the two layers is pressed between the forging and shear punch after the shear die is removed. Finally, the deformed sheared part produces a force- and form-closed joint. Pedreschi et al. [17] introduced a press-joining process with a rectangular mold for cold-formed steel structures. They also studied the influences of shear test direction, joint number, raw material thickness and strength, shearing edge width, and different thickness combinations. Davies et al. [18] investigated the moment-rotation behavior of a set of rectangular joints in cold-formed steel structures. Raw metal thickness and joint arrangement have an important effect on moment capacity and rotation characteristics. Davies et al. [19] further studied the shear behavior of rectangular joints and derived a formula for the prediction of joint peak load. They found that joint peak load increases as raw material thickness and strength are increased. The maximum shear test load is obtained when the shear test direction is perpendicular to the long edge of the rectangular joint. The stiffness of the load–joint movement curve gradually reduces from the perpendicular direction to the parallel direction. Varis [20,21] compared the performances of high-strength structural steel joints formed with round molds with those of high-strength structural steel joints formed with rectangular molds, and found that although the maximum shearing load of round joints is greater than that of square joints, the deformation capacity of the latter is greater than that of the former. Pedreschi and Sinha [22] derived an expression for joint strength through theoretical prediction based on two direction failure modes and analyzed the theoretical estimation based on two vertical shear failure modes. Pedreschi and Sinha [23] reported that the joint number has a considerable influence on the strength, deformation, and failure mode of cold-formed steel trusses. This finding proves that rectangular joints can be effectively utilized for truss connection. Gronostajski and Polak [24] analyzed the quasistatic and dynamic deformation of double-hat thin-walled elements joined through round and rectangular press joining and showed that both kinds of mechanical joining can be applied to controlled-body crushing zones. Mucha [25] studied the detailed influence of layout angle on rectangular joint performance. They found that the energy dissipation and maximum shearing force when the layout angle was 90° is 50% and 40% higher, respectively, than those when the layout angle was 0°. Large material locks have a crucial effect on joint rigidity under lateral joint loads. Lambiase [26] applied different molds to produce polymer–metal hybrid joints and showed that rectangular molds require the lowest joint force and provide the maximum peel strength but have poor shear strength because of surface damage. Abe et al. [27] utilized rectangular shear clinching to join ultra-high-strength steel. In this process, the upper plate is locally sheared, and the lower plate remains intact. Their results showed that the strength of steel joints produced through rectangular shear clinching was one-third of that of welded ultra-high-strength steel joints.

The development of small devices increases the demand for joining processes of different kinds of lightweight metal foils in the micro dimension. However, the mechanical clinching process is normally applied on metal plates with thicknesses of 0.2 to 4 mm and is seldom used to join metal foils with thicknesses below 0.2 mm. Due to size effect [28], the traditional mechanical clinching process cannot be directly imported into metal foil joining. In addition, the difficulty and high cost of fabricating suitable micro tools need to be considered. The small clearance between the micro punch and die makes it difficult to guarantee the alignment accuracy and impairs micro tool service life and joint quality. The laser shock forming process is a non-contact loading technology which can avoid and replace the micro punch and is advantageous for plastic deformation in the micro field. Thus, researchers are trying to combine laser shock forming and traditional mechanical clinching to develop micro clinching of metal foils. Ji et al. [29] published a patent on micro foil clinching, where laser shock makes the upper foil distribute into the pre-drilled hole of the lower foil, forming an S-shape mechanical lock. Weilage and Vollertsen [30] produced an undercut in the micro range for a single aluminum foil with thicknesses of 20 and 50 μm by 20 or 50 laser shocks, which directly proved that laser shock forming could deform undercuts of metal foil thinner than 200 μm in the micro dimension. In order to make each laser pulse fully promote the foil forming for micro joining, Veenaas et al. [31] measured acting pressure in near-by field of shockwaves in an open and tube environment, which determined that the ignition point of the plasma induced by a TEA-CO_2_ laser is about 8 mm above the aluminum surface. Based on the former results, Veenaas and Vollertsen [32,33] successfully applied laser shock forming on metal foils joining in the micro range with a pre-drilled hole on the lower stainless-steel foil and with 50 laser pulses. With confinement and an ablative-layer-assisted laser forming process, Wang et al. [34] also realized metal foil joining by dozens or hundreds of laser pulses for annealed upper copper foil and perforated lower stainless-steel foil. Additionally, Wang et al. [35] utilized laser shock forming to join a three sheet combination of two annealed upper copper foils and one perforated lower stainless-steel foil. Different from the above pre-drilled hole joining process, Wang et al. [36] proposed a novel process of micro clinching with cutting by laser shock forming. A special die can cut out a hole on the lower foil and realize the joining synchronously with only three laser pulses, but the process is limited because the upper foil should be thicker than lower foil. In addition, Wang et al. [37] determined the detailed influence of process parameters of the micro clinching for further application.

However, the above realized micro mechanical joining by laser shock forming all need a material removal process and has a complicated joint deformation requirement. The application of processes is also limited by too many laser pulses or thickness combination requirements, which is detrimental to the application of micro joining by laser. Therefore, this study proposes a micro-shearing clinching that applies three laser pulses and bypasses the material removal procedure. The process draws on rectangular shear clinching and micro joining by laser shock forming. The process is simplified because the alignment requirement for the laser in the length direction of the mold is considerably lower than that for the laser in the width direction.

This paper first studies the determination of basic process parameters and investigates the joint deformation process to improve our understanding of micro-shear clinching; then the process windows and mechanical properties of similar material combinations are acquired for basic application of the process; finally the process is successfully introduced to joining different material combinations.

## 2. Mechanism and Experimental Setup of Micro-Shear Clinching

**Figure 1:**
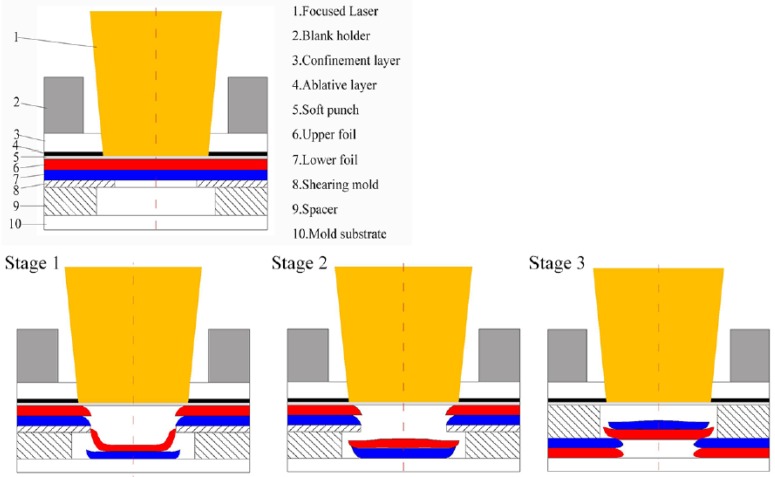
Schematic diagram of micro-shear clinching by laser shock.

The schematic of the micro-shear clinching by laser shock is shown in Figure 1. It was composed of a focused laser, blank holder, confinement layer, ablative layer, soft punch, upper foil, lower foil, shearing mold, spacer, and mold substrate. The laser beam first irradiated the ablative layer through the transparent confinement layer. Then parts of the ablative layer immediately absorbed a large amount of energy and transformed into a high-temperature and high-pressure plasma. The confinement layer constrained the upward expanding of the plasma. Then the downward expanding of the plasma induced a large shockwave. The soft punch was impacted by the induced laser shockwave, and then squeezed into the combined shearing die. Finally, the specimen was deformed or sheared into a certain shape.

The micro-shear clinching process can be divided into three main stages: (1) drawing and shearing; (2) flattening; and (3) re-striking and fitting, as shown in Figure 1. In Stage 1, when the laser shock is applied on the upper foil side, the drawing of materials occurs; then with the collective actions of shearing edges and mold substrate, the drawing part is finally sheared along the two shear edges raising bumps on two foils. In Stage 2, the laser shock is applied on the upper foil side to flatten the sheared part of two foils with the support of mold substrate. In Stage 3, the shearing mold and spacer are removed; relative displacement between the upper and lower foils does not occur because the joint is preliminarily deformed, then the preliminary joint is turned over and stacked with a thicker spacer; then by applying same laser shock forming process on the lower foil, the sheared part is re-struck and fits on the notch of the raw foils; subsequently a form- and force-closed joint is deformed.

**Figure 2:**
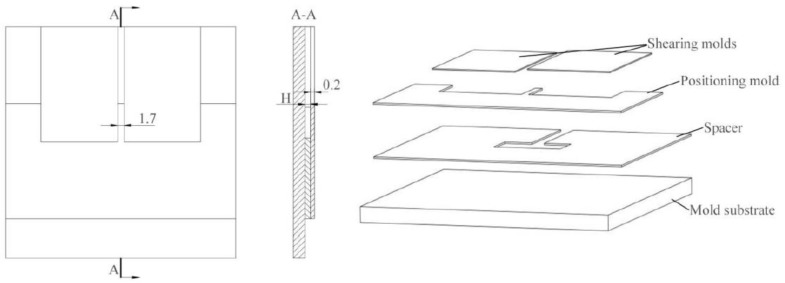
Primary designed and combined rectangular shearing mold.

The laser beam with Gaussian distribution was generated by a Nd-YAG (neodymium-doped yttrium aluminum garnet; Nd: Y_3_Al_5_O_12_) Spitlight 2000 laser (INNOLAS Corporation, München, Germany). The main parameters of the laser are listed in Table 1. The applied laser spot had a diameter of 2 mm and was focused via convex lens. The blank holder provides 12 N of pressure. The confinement layer was made of highly transparent PMMA (polymethyl methacrylate) with a thickness of 3 mm. Black paint was used as an ablative layer and uniformly sprayed on the confinement layer, and its thickness was approximately 10 μm. Silica gel was adopted as the soft punch. To maximize the load of laser shock, the confinement layer with black paint was renewed and the soft punch was replaced for every laser pulse. As shown in Figure 2, the primary combined rectangular shearing mold comprises four adjustable components that include positioning mold, shearing mold, spacer, and mold substrate. The positioning mold was used to limit shearing mold movement and determine the width between two shearing edges of two shearing molds. The thickness of the shearing mold was 0.2 mm, which is stiff enough to meet the requirement. The width between the two shearing edges of the two shearing molds is 1.7 mm. H represents the thickness of the spacer at Stage 2; the spacer in Stage 3 was decided by the spacer in Stage 2, which was 200 μm thicker than the spacer at Stage 2. The spaces in the middle of the spacers were sufficiently large to accommodate foil deformation. The mold substrate was used to limit the vertical deformation of foils.

A single-factor experimental design was adopted to investigate the main effect of the process parameters to have a fundamental understanding of the process. The constant parameters for all experiments are listed in Table 2. The other parameters are listed in the corresponding research section. Two types of metal foils were employed in the investigation. The foils included 1060 pure aluminum foil (Al), and pure pure T2 copper foil (Cu); “Al/Cu” indicates that the Al foil is the upper foil and Cu foil the lower foil; “100/60 μm” indicates that the thickness of the upper foil is 100 μm and the thickness of lower foil is 60 μm.

**Figure 3:**
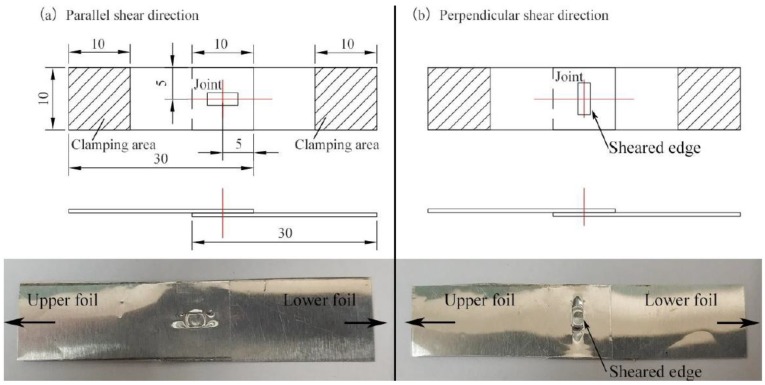
Dimensions and two shear directions of the single-lap shearing test sample.

The mechanical strength of each joint was measured by a single-lap shearing test for two shear directions, which are parallel and perpendicular to the sheared joint edge. Figure 3 presents the dimensions and two shear directions of the single-lap shearing test sample. All samples were tested using an electronic universal testing machine (Instron Type UTM 4104, Shenzhen, China). The test was conducted at room temperature with a constant speed of 2 mm/s.

## 3. Results and Discussion

### 3.1. Determination of Three Basic Process Parameters

Micro-shear clinching involves three basic process parameters for laser shock forming: soft punch thickness and number of laser pulses on the upper and lower foil sides. Normally, basic process parameters are constant for joining different materials, which would directly and greatly influence the joint deformation and efficiency of process. Thus, the determination of these three basic process parameters is important. In this section, the combination of Al/Al with different basic parameters was experimentally tested in order to obtain a better understanding and determination of the three basic process parameters.

#### 3.1.1. Determination of Soft Punch Thickness

The employment of the soft punch ensures good joint surface quality, but different soft punch thicknesses will influence the joint deformation. The experiments investigated four different thicknesses of soft punch to determine the suitable thickness. The experimental process parameters are listed in Table 3.

**Figure 4:**
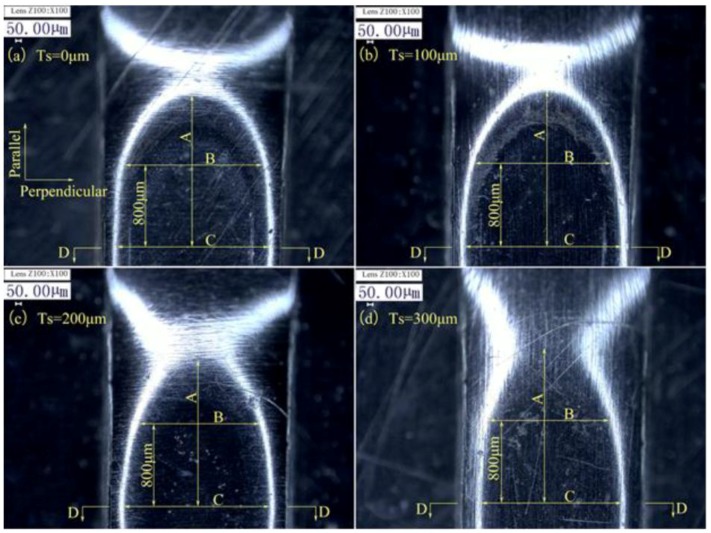
Lower foil side-view of the preliminary joints with four different soft punch thicknesses: (**a**) Ts = 0 μm; (**b**) Ts = 100 μm; (**c**) Ts = 200 μm; (**d**) Ts = 300 μm.

**Figure 5:**
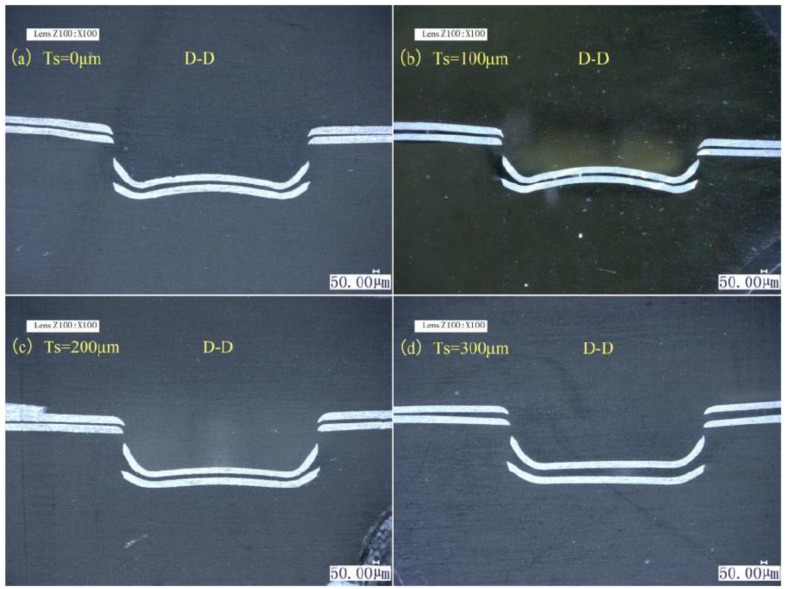
D–D section of preliminary joints with four different thicknesses of the soft punch: (**a**) Ts = 0 μm; (**b**) Ts = 100 μm; (**c**) Ts = 200 μm; (**d**) Ts = 300 μm.

**Figure 6:**
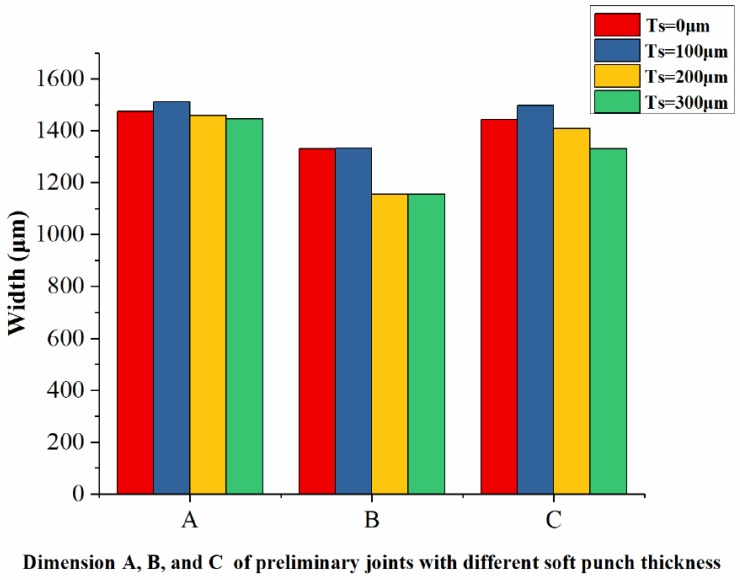
Dimension A, B, and C of preliminary joints with four different soft punch thicknesses.

The lower foil side-view and cross-sections of the four preliminary joints produced with four different soft punch thicknesses are shown in Figure 4 and Figure 5. In Figure 4, C is the dimension of the joint on the perpendicular symmetry plane that was perpendicular to the sheared edge; B is the dimension of the joint on the plane that was 800 μm away from the perpendicular symmetry plane; A is the dimension of the joint on the parallel symmetry plane which was parallel to the sheared edge; D–D is the cross-section on the perpendicular symmetry plane. The half preliminary joint was measured from the lower foil side, as shown in Figure 6, in consideration of the process symmetry.

Figure 4 and Figure 6 show that when soft punch thickness is increased from 100 to 300 μm, the formation area of preliminary joint narrows and the three dimensions gradually decrease. The maximum joint formation in Dimension A was obtained under a soft punch thickness of 100 μm. The second-highest joint formation was produced under a soft punch thickness of 0 μm (without soft punch). The joint formation in Dimension B under the soft punch thickness of 100 μm was slightly greater than that under the soft punch thickness of 0 μm. The joint formations with the two other soft punch thicknesses were similar and considerably smaller than those with the former two soft punch thicknesses. The soft punch thickness of 100 μm also produced the maximum joint formation in Dimension C. The trend shown by the joint formation in Dimension C was the same as that shown by the joint formation in Dimension A. This finding shows that the soft punch thickness of 100 μm can optimize the use of each laser pulse to maximize deformation. Wang et al. [38] explained that when the laser shockwave first acts on a soft punch with low impedance and propagates from the soft punch into thin metal foils with high impedance, the impedance mismatch effect will increase the shockwave pressure. Hence, the use of the soft punch can improve process efficiency and reduce the potential number of laser pulses. Wang et al. [37] explained that because of the increased speed of the unloading wave in the soft punch, the unloading wave will catch up with the elastic wave until it propagates into the thin metal foils with high impedance when the soft punch is excessively thick. Hence, the final stress on the workpieces decreases. In contrast to the soft punch thicknesses of 0, 200, and 300 μm, a soft punch thickness of 100 μm can promote joint deformation.

In Figure 5, upper and lower foils were subject to drawing and shearing actions. The 100 μm soft punch produced the greatest spring back, and when soft punch thickness increases from 100 μm to 300 μm, the spring back gradually diminished and even disappeared. Increased joint formation and intensive spring back are produced by increased shockwave pressure synchronously. The results for the D–D section show that all of the four kinds of soft punches can successfully cause sheared parts to separate from raw foils. Moreover, spring back can be vastly reduced by the subsequent flattening action.

Therefore, a soft punch thickness of 100 μm can improve laser shock formation and efficiency by magnifying shockwave pressure. The process and the following experiments all employ a soft punch thickness of 100 μm.

#### 3.1.2. Determination of Number of Laser Pulses on the Upper Foil Side

Micro-shear clinching first requires laser shocks on the upper foil side. The laser pulses on the upper foil side exert drawing, shearing, and flattening actions on the preliminary joint. These actions serve as the foundation for the re-striking action of laser pulses on the lower foil side. The number of laser pulses on the upper foil side should be minimized to ensure process efficiency and sufficient joint formation. Three different levels of laser pulse numbers were experimentally investigated. The experimental process parameters are listed in Table 4. The laser energy for upper foil side is En_1_.

**Figure 7:**
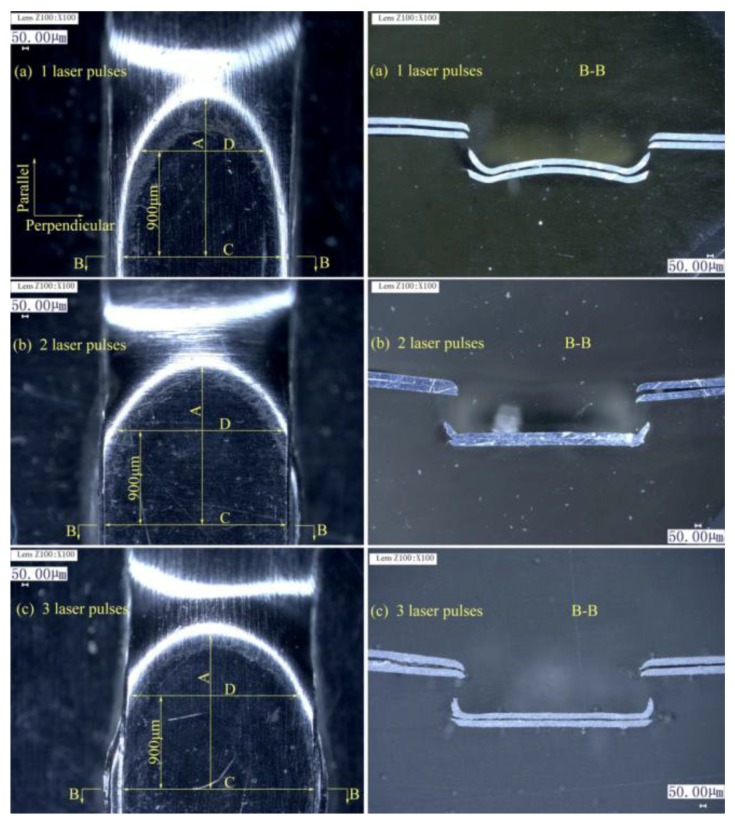
Lower foil side-view and B–B section of preliminary joints with three different numbers of laser pulses on the upper foil side: (**a**) 1 pulse; (**b**) 2 pulses; and (**c**) 3 pulses.

**Figure 8:**
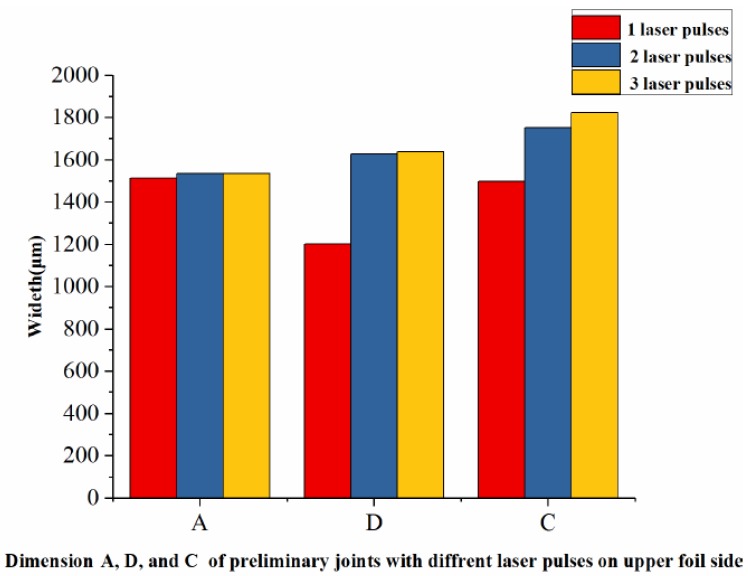
Dimensions A, D, and C of preliminary joints with three different numbers of laser pulses on the upper foil side.

The laser energy applied on the upper foil side was 1380 mJ. The lower foil side view and cross-sections of the preliminary joints with three different numbers of laser pulses on the upper foil side are shown in Figure 7. The measurement of the half of the lower foil side of the preliminary joint is shown in Figure 8.

In Figure 7, C is the dimension of the joint on the perpendicular symmetry plane which was perpendicular to the sheared edge; D is the dimension of the joint on the plane that was 900 μm away from perpendicular symmetry plane; A was the dimension of the joint on the parallel symmetry plane that was parallel to the sheared edge; B–B was the cross-section on the perpendicular symmetry plane.

Joint formation in Dimension A under two laser pulses was similar to that under three laser pulses. These two formations were both greater than joint formation under one laser pulse. Joint formation in Dimension D with three laser pulses was slightly greater than that under two laser pulses, and these two formations were considerably greater than formation under one laser pulse. Joint formation in Dimension C showed remarkable increments as the numbers of laser pulses were increased from 1 to 3. Hence, the numbers of pulses improve joint formation in the laser loading edge area by a limited extent but have a remarkable effect on joint formation in the center when joint formation reaches a certain degree. Although the work hardness and mold limit reduce formation, the energy density on the laser center was high. Moreover, the perpendicular boundaries of the joint center were less limited than those of other areas and promoted formation. The use of two and three laser pulses could induce sufficient integral deformation in the joint, and the effects of using two laser pulses do not significantly differ from those of using three laser pulses. Additionally, formation could be improved by increasing the energy of every laser pulse. Hence, the use of two laser pulses on the upper foil side is suitable for the process and has a higher efficiency than the use of three laser pulses.

In Figure 7, section B–B reflects formation on the perpendicular symmetry plane. The use of one laser pulse ensues joint drawing and shearing. However, the width of the sheared part does not exceed the notch on raw foils. The use of two and three laser pulses can flatten the sheared part and eliminate spring back and ensures that the sheared part is larger than the notch. The use of two and three pulses is distinguished by the formation of sharp angles on both sides of the sheared part. The sharp angles that form under two pulses follow an oblique outward orientation because the second pulse flattens the sheared part after the first pulse, and the force borne by the edge of sheared part was less than that borne by the middle region. Meanwhile, the sharp angles of the sheared part produced under three pulses bend inward when the third laser pulse was applied on the flattened sheared part produced by two laser pulses, and the laser shock directly strikes the flattened sheared part, causing outward material flow that then leads to the bending of inward angles. The results for the B–B section show that the use of two and three laser pulses can meet process requirements.

Therefore, two laser pulses on the upper foil side can induce the sufficient joint formation and ensure process efficiency. In the process, laser energy of determined laser pulses can be adjusted to meet different deformation requirements of different materials. Then, the experiments of the number of laser pulses on the lower foil side were conducted.

#### 3.1.3. Determination of the Number of Laser Pulses on the Lower Foil Side

The laser pulses on the lower foil side exert a re-striking action on the preliminary joint. Two different numbers of laser pulses were investigated experimentally. The experimental parameters are listed in Table 5. The laser energy for the lower foil side is indicated as En_2_. The energy of laser pulses on the lower foil side was smaller than that on the upper foil side, because the sheared part had a little limit from the combined mold and was easy to be re-struck.

**Figure 9:**
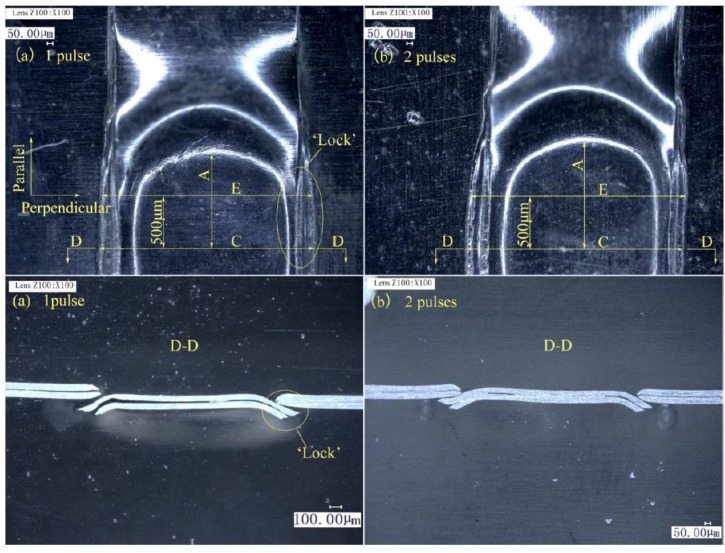
Lower foil side view and D-D section of joints with two different number of laser pulses on the lower foil side: (**a**) 1 pulse; (**b**) 2 pulses.

**Figure 10:**
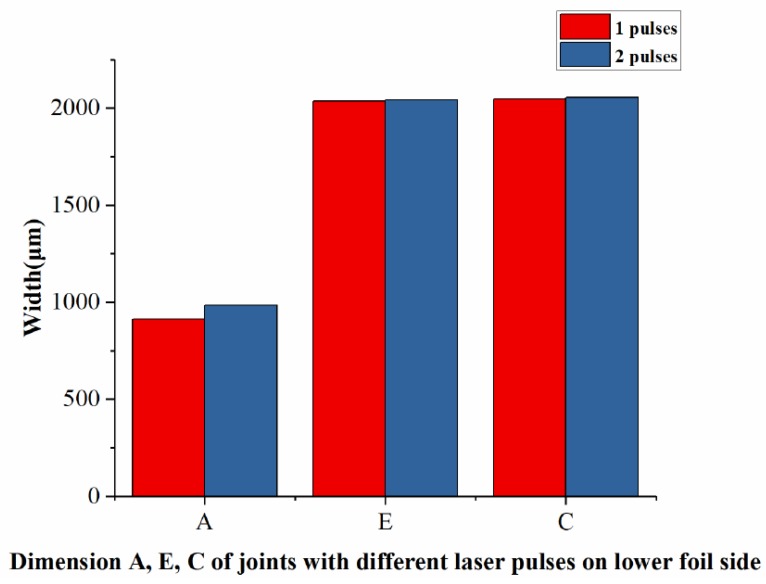
Dimensions A, E, and C of joints with two different numbers of laser pulses.

The lower foil side-view and cross-sections of the two joints with two different numbers of laser pulses on their lower foil side are shown in Figure 9. The measurement of the half lower foil side of the joints is shown in Figure 10. In Figure 9, C is the dimension of the joint on the perpendicular symmetry plane that was perpendicular to the sheared edge. E is the dimension of the joint on the plane that was 500-μm away from the perpendicular symmetry plane. A is the dimension of the joint on the parallel symmetry plane that was parallel to the sheared edge. D–D is the cross-section on the perpendicular symmetry plane.

Figure 9 and Figure 10 show that the two kinds of laser pulses enable joining. Moreover, the extent of joint formation under two laser pulses on the lower foil was greater than that under one pulse. Joint formation in Dimension A under two laser pulses was greater than that under one laser pulse. For Dimensions E and C, the joint formation under two laser pulses and on laser pulse were similar. The results show that the increase in the laser pulses on the lower foil side from one to two had a greater effect on formation along the parallel direction rather than perpendicular direction of the joint when the laser energy was small. In the perpendicular direction, the first laser pulse had already re-struck and flattened the sheared part on the raw foils. The second laser pulse simply provides the flattening action, which had a limited effect on the improvement in formation because of the reduction in energy. However, in the parallel direction after the first laser shock, the sheared part still needed to be re-struck and flattened. Thus, formation in the parallel direction was more sensitive than that in the perpendicular direction. The results shows that the second laser pulse had a limited improvement for the Dimensions E and C. Because the formation is sensitive to the increase in laser pulses owing to the small laser energy, and two laser pulses bring limited improvement, one laser pulse on the lower foil side is suitable for the process. The formation improvement can also be achieved by increasing laser energy instead of laser pulses. In Figure 9, The D–D section reflects the formation of “Lock” on the perpendicular symmetry plane. The two kinds of laser pulses all successfully completed the joining.

The optimal number of laser pulses on the lower foil side was determined to be 1. The use of one laser pulse could meet different material deformation requirements given that it will improve energy disunity in the entire loading area.

For joint deformation and efficiency requirements of the process, the three basic process parameters are determined based on the above researches, and all the following experiments employ these basic process parameters as listed in Table 6.

### 3.2. Joint Deformation Process

Joint deformation can be divided into three stages in accordance with the extent of joint deformation caused by the laser pulses on the upper and lower foils, which also are in accordance with the three stages of micro-shear clinching. Two laser pulses on the upper foil side stage corresponded to Stages 1 and 2. One laser pulse on the lower foil side corresponded to Stage 3. Accordingly, as described in this section, the Al/Al combination was used to determine the detailed formation behavior and thickness distribution presented by the upper and lower foils during the three stages. The laws governing deformation can provide a basic understanding of this process to acquire qualified joints. The experimental parameters are listed in Table 7.

**Figure 11:**
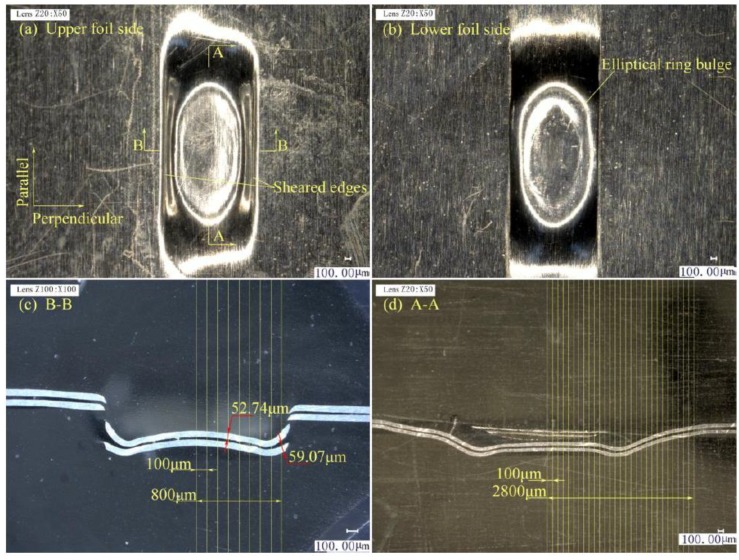
Preliminary joint formation in stage 1: (**a**) Upper foil side appearance; (**b**) Lower foil side appearance; (**c**) B–B section on perpendicular symmetry plane; (**d**) A–A section on parallel symmetry plane.

**Figure 12:**
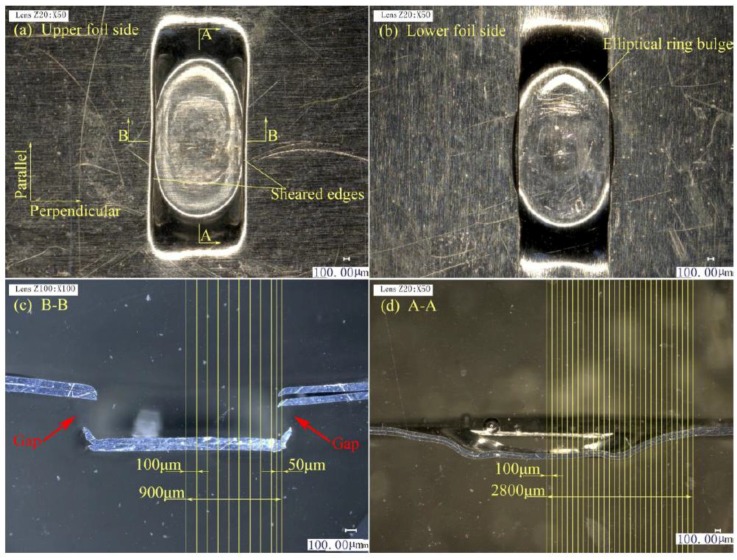
Preliminary joint formation in Stage 2: (**a**) upper foil side appearance; (**b**) lower foil side appearance; (**c**) B–B section on perpendicular symmetry plane; and (**d**) A–A section on parallel symmetry plane.

**Figure 13:**
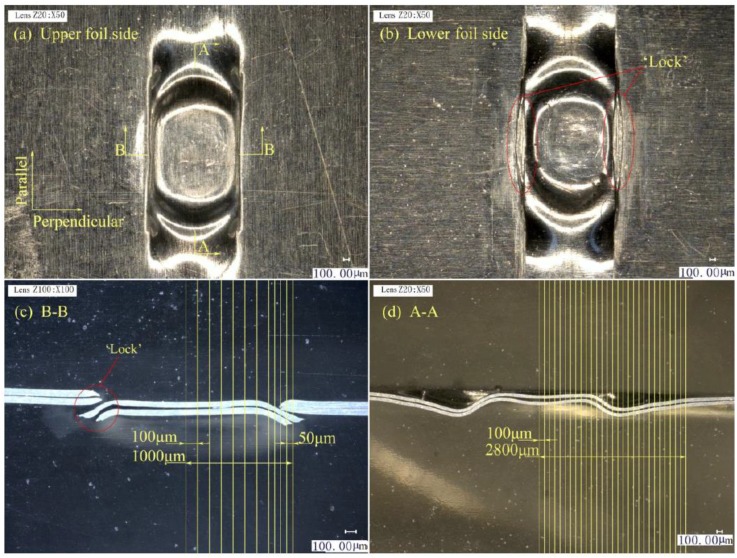
Joint formation in Stage 3: (**a**) upper foil side appearance; (**b**) lower foil side appearance; (**c**) B–B section on perpendicular symmetry plane; and (**d**) A–A section on parallel symmetry plane.

The upper and lower foils and the two joint cross-sections in three stages are illustrated in Figure 11, Figure 12 and Figure 13. A–A is the cross-section along the parallel symmetry plane of the joint. B–B is the cross-section along the perpendicular symmetry plane of the joint.

In Stage 1, as shown in Figure 11a, the formed joint has an elliptical shape, and the sheared port diffuses along the elliptical shape boundary, because the laser energy has Gaussian distribution. Figure 11b shows that the bottom of the sheared part has an elliptical bulge caused by the spring back phenomenon in the middle part of the joint. The elastoplastic property of the metal foils causes the spring back. Figure 11c presents that laser shock which causes the drawing and shearing of the upper and lower foils in the B–B section because of the low depth-to-width ratio and shearing edges of the mold. The upper foil was stacked on the lower foil, and the lower foil was stacked on the shearing mold. Thus, the lower foil was firstly sheared by the shearing edges, and the drawing and necking of the lower foil were less than those of the upper foil. The drawing part of the upper foil was the key to “Lock” formation. However, as shown in Figure 11d, the drawing of the two foils was highly uniform along the parallel direction because fewer limits existed along the parallel direction than the perpendicular direction of the combined mold. Pronounced spring back also appeared in the A–A section.

In Stage 2, as shown in Figure 12a, the elliptical joint expands along the parallel direction. In contrast to that in Stage 1, the sheared port elongates along the sheared edge in the parallel direction in Stage 2 because of the low limit of the combined mold on metal foils along the parallel direction. The elliptical ring bulge on the sheared part in Figure 12b became larger than that in Figure 11b, and the overall sheared part was flatter than in Stage 1. The width of the sheared part exceeded that of the shearing edges because the drawing and sheared part was flattened. Subsequently, they became longer than the notch of the raw foils. In Figure 12c, the two foils were tightly stamped together by laser shock because the shockwave was limited by the width of the shearing edges in the perpendicular direction. The bottom of the sheared part had a flat surface. The edges of the stamping area were tilted up because of the thinning deformation of the stamping area. The edges of the stamping area were tilted upward because of the thinning deformation of the stamping area. A gap always exists between the sheared part and raw foils with flattening actions on the sheared part. As the laser energy decreases from the center to the edge, the gap also decreases starting from the B–B section. Joining is based on the existence of the gap. As shown in Figure 12d, the two foils are also tightly stamped together. Spring back continues to exist but has been reduced.

In Stage 3, as shown in Figure 13a, the sheared part was re-struck and tightly fits the notch of the foils. In Figure 13b, the joint was formed and the “Lock” appears on the lower foil side and along the two sheared edges. In Figure 13c, the sheared part was re-struck back to the notch. Although the laser spot was greater than the sheared part, the notch with a certain depth and laser energy with Gaussian distribution will cause the edges of the sheared part to tilt down. The thinning of the center of the sheared part was smaller than the edges, because the edges of sheared part were re-struck on the notch edges. These edges were under local drawing actions. The formed sheared parts of the upper foil directly join the two foils, and the formed sheared parts of the lower foil exert a stamping action on the upper foil. In Figure 13d, the two foils continue to exhibit different extents of spring back. The upper foil, however, was flatter than the lower foil.

Therefore, during the whole process, the two foils were continuously deformed through drawing, shearing, and restriking without forming complicated shapes.

**Figure 14:**
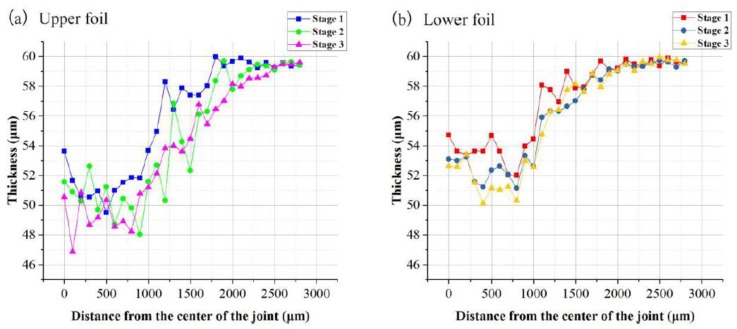
The A–A section thickness distribution curves in three stages: (**a**) upper foil and (**b**) lower foil.

**Figure 15:**
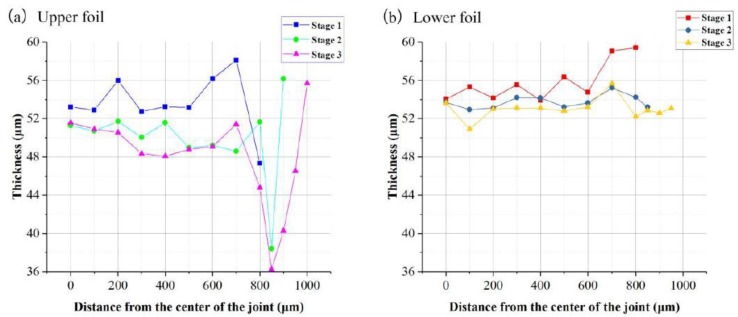
The B–B section thickness distribution curves in three stages: (**a**) upper foil and (**b**) lower foil.

**Figure 16:**
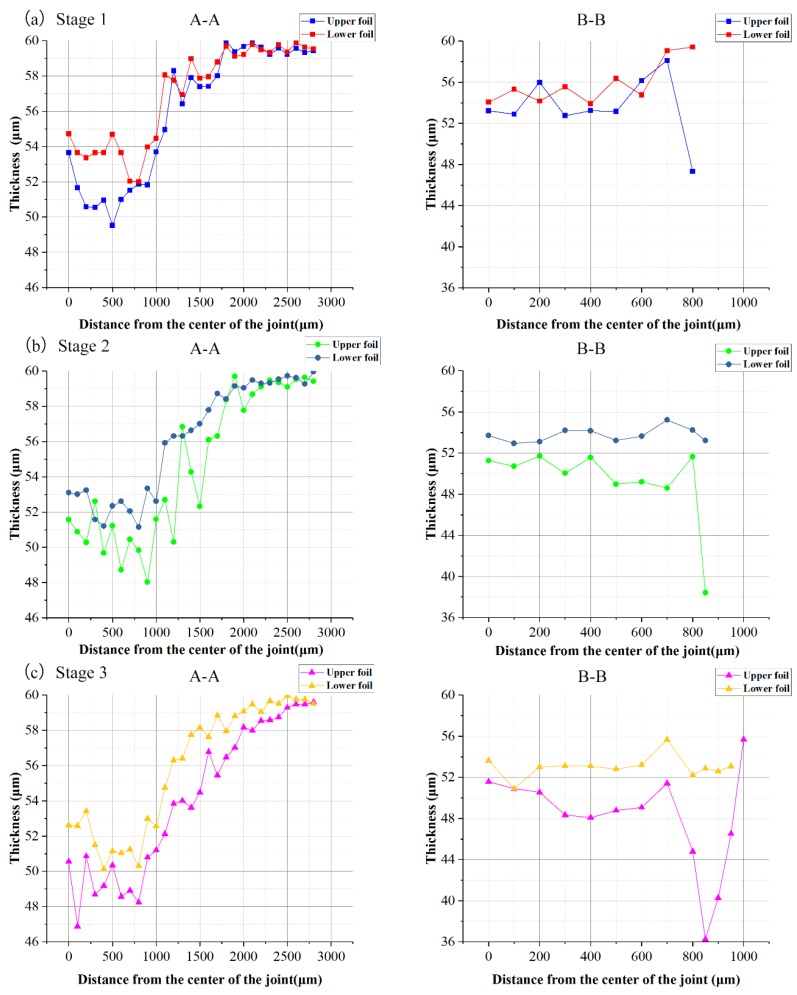
Thickness distribution comparisons of upper and lower foils: (**a**) Stage 1; (**b**) Stage 2; (**c**) Stage 3.

The thicknesses of the right halves of the A–A and B–B sections were taken to evaluate the overall thickness distribution of the two foils at the three stages. The measurement distance begins from the dotted symmetric centerline and increased by 50 μm or 100 μm. The measurement locations are marked with yellow lines. Every thickness measurement was taken starting from the intersection of the yellow line and the upper surface of each foil along the vertical tangential direction. Two thickness measurement samples are marked in red in Figure 11c. The thickness distribution curves for the A–A and B–B sections of the two foils at the three stages are presented in Figure 14 and Figure 15. The comparison of the thickness distribution of upper and lower foils for every stage is shown in Figure 16.

The evolution of the overall thickness distribution in the A–A section was acquired as illustrated in Figure 14. The thicknesses of the two foils continuously decreased from Stage 1 to Stage 3. Thickness first decreased from the joint center to the edge and then increased to raw foil thickness. Although the laser energy in the joint center was the highest, the thinnest part was located next to the center. This thickness distribution pattern was the same as that observed in the other drawing processes and developed because the center part was subject to large compressive stress rather than tensile stress. Moreover, high tensile stress acts on the area next to the center. In contrast to that at Stages 2 and 3, laser energy provided the major contribution to the deformation of the two foils at Stage 1 because foil deformation was less restricted and work hardness had not yet appeared in this stage. The thickness distribution patterns at Stages 2 and 3 show negligible differences because the restruck laser energy at Stage 3 was relatively small and work hardness reduced the effect of the laser shockwave. As shown in Figure 14b, the thickness evolution of the lower foil negligibly differed across the three stages in contrast to that of the upper foil shown in Figure 14a given that the drawing action on the lower foil was negligible after the lower foil was first sheared. Then, re-striking and flattening exerted a weak thinning effect on the lower foil.

As shown in Figure 15, the overall thickness of the two foils continuously decreased from Stage 1 to Stage 3 in the B–B section. The thinnest part of the upper foil was close to the joint edge because of the shearing action. However, the lower foil had uniform thickness and lacked an observable thin part because it first undergoes shearing with fewer drawing actions than the upper foil. The thinnest part of the upper foil was located approximately 850 μm away from the joint center at every stage. It approaches the horizontal distance of the shearing edge of the shearing mold because of the drawing action at Stage 1. It was also located at the boundary of the sheared part at Stage 2. In addition to the joint formation induced in the former stage, the lower foil of the sheared part was shorter than the upper foil at the boundary. This characteristic results in the occurrence of a local drawing effect on the upper foil. The sheared edge of the raw foils also exerted a local drawing effect that induced the thinning of the upper foil at Stage 3. Finally, the deformed foil part over a 850 μm distance became the “Lock” of the joint.

Therefore, the laser pulse provided the major contribution to the deformation of the two foils at Stage 1. The thicknesses of the two foils continuously decreased over Stages 1 to 3. The thickness distribution patterns of the two foils, however, are not considerably different at Stages 2 and 3. These results indicate that the first laser pulse had a drastic influence on joint formation, and the third laser pulse did not cause pronounced foil thinning. The thickness in the A–A section first decreased from the joint center to the edge and then increased to raw foil thickness. In addition, the thinnest part was located next to the center. The thinnest part of the upper foil in the B–B section was located near the joint edge, and the thickness of the lower foil was uniform.

In Figure 16a, in the A–A section, the thickness difference shown by the joint center was larger than shown by the joint edge. However, in the B–B section, the thickness difference was noticeable only at the edge. Overall, the thickness of the upper foil was larger than that of the lower foil. Because the upper foil was sheared after the lower foil was sheared, which caused more tensile stress in upper foil.

In Figure 16b, compared with that in Figure 16a, the integral thickness difference in the A–A section was reduced in the tcenter, because the two foils fit more tightly and spring back in the center of the joint was reduced. In the B–B section, the closer the location was to the joint edge, the greater the thickness difference was observed between the two foils. Because the joint edge in the B–B section was similar to the free end with little limit, which was easier to form.

In Figure 16c, the thickness difference between the upper and lower foils is pronounced at this stage. Although the laser energy remains low at this stage, the two foils undergo joint formation because they are restruck to fit the raw metal foils, wherein the sheared part is free and easily undergoes joint formation.

Therefore, the higher tensile stress borne by the upper foil than that borne by the lower foil causes the upper foil to become thinner than the lower foil during the whole process.

The law that governs joint formation by the upper and lower foils at the three stages indicates that the employed foils must have a certain degree of plasticity. Joint deformation mainly includes drawing, shearing, and restriking and lack complicated deformation requirements. Laser shocking is a rapid manufacturing process that facilitates the production of shearing action. Thus, thin plates made of special materials, such as plastics and composites, can also be employed in laser shocking. The deformation of the upper foil is greater than that of the lower foil. Thus, materials with good plasticity can be used as the upper foil, and other materials can be used as the lower foil.

### 3.3. Process Windows and Joint Characteristics of Similar Materials

#### 3.3.1. Process Windows of Al/Al

Three other influential process parameters, namely, laser energy (En_1_) for Stage 1 and Stage 2, laser energy (En_2_) for Stage 3, and spacer thickness (H), will affect the joinability of different foil combinations in addition to the determined basic process parameters. To acquire the reasonable range of process parameters for different thickness combinations, the influences of the above three process parameters on joinablity of different thickness combinations were investigated. The experimental process parameters are listed in Table 8. Experimental results showed that Al/Al combinations with same total thickness have the same required process parameters. Three process windows of Al/Al by micro-shear clinching were acquired, which was related with these three influential process parameters.

**Figure 17:**
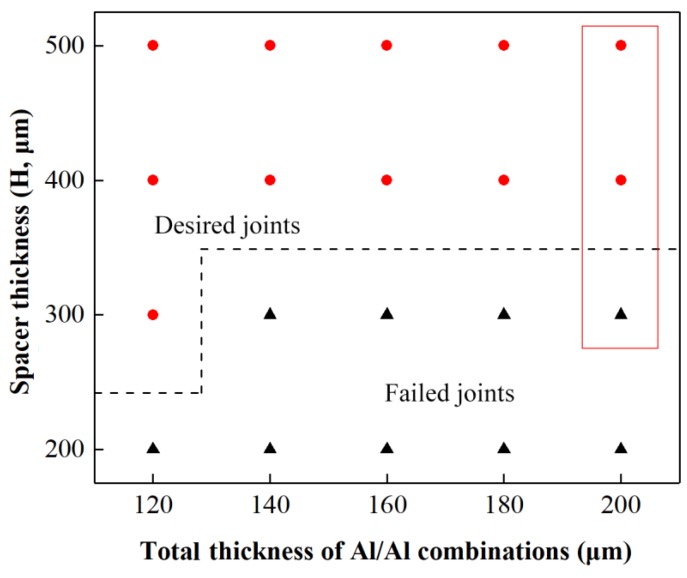
Process window of total thickness with H for Al/Al combinations.

**Figure 18:**
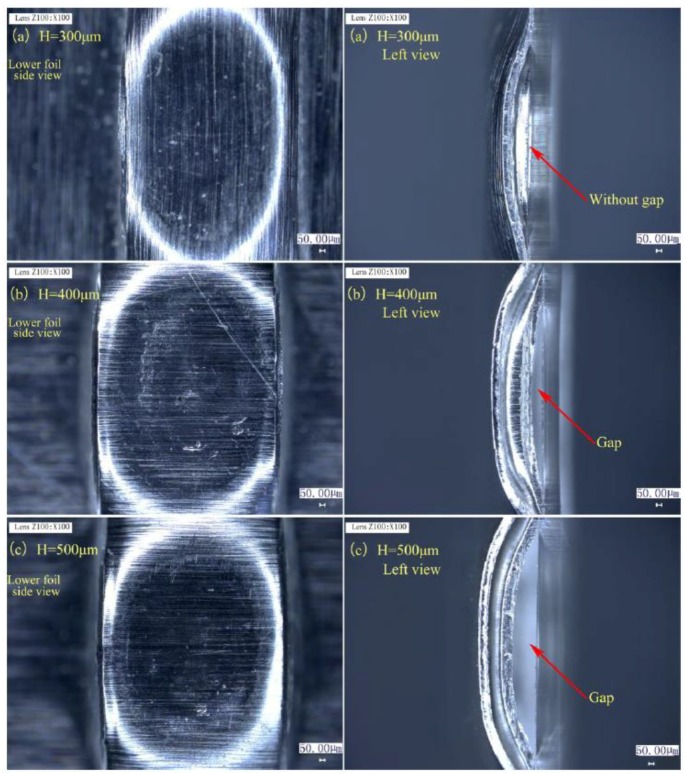
Preliminary joints produced under En_1_ of 1690 mJ and three different H: (**a**) H = 300 μm; (**b**) H = 400 μm; (**c**) H = 500 μm.

Figure 17 shows the matching relationship between spacer thickness H and total thickness, in which En_1_ is adjusted from 515 to 1800 mJ for every spacer thickness in the H and total thickness combination. Figure 17 was acquired by studying preliminary joints formed in Stage 2. The desired joint part shows that the drawing and shearing actions for preliminary joint with certain H values are feasible for acquiring the final joint. The failed joint part indicates that certain qualified preliminary joints cannot be formed under certain H values even if the optimal laser energy is used. The results illustrate that spacer thickness H must be at least twice the total thickness. Moreover, when high H values are employed for thin combinations, joining with increased deformation and without ruptures is achieved because of free deformation along the length of the combined mold and the good plasticity of Al foils. The preliminary joints of 100/100 μm produced by different spacers with H values of 300, 400, and 500 μm and En_1_ of 1690 mJ are presented for comparison in Figure 18. These joints belong to the points marked by red rectangles in Figure 17. From the lower foil side view of the three joints in Figure 18, an insufficient and asymmetrical formation was produced in the lower foil under an H of 300 μm despite the employment of high laser energy. However, the H of 400 and 500 μm produces the sufficient and asymmetrical formation of preliminary joints because when the spacer is sufficiently thick, the considerable plastic deformation presented by metal foils can reduce or offset the misalignment and non-uniformity of the metal foils. From the left view of three joints in Figure 18, the preliminary joint produced by H of 300 μm did not form a suitable gap. This result indicates that the sheared and formed part of the upper foil cannot be restruck on the lower foil at Stage 3 and finally causes joining failure. Shearing actions can produce suitable gaps only when two foils are thinned to a certain degree by drawing actions. The employment of thick spacers can enhance drawing and shearing actions under the same conditions. Therefore, H has a fundamental influence on joint formation and must be increased as total thickness is increased to accommodate additional spaces for the drawing and shearing actions of metal foils. This requirement cannot be achieved by simply increasing laser energy.

**Figure 19:**
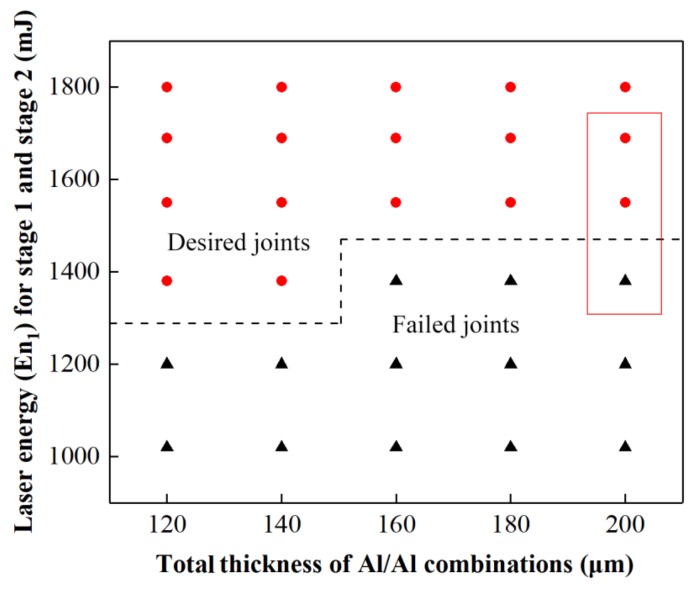
Process window of Total thickness with En_1_ for Al/Al combinations.

**Figure 20:**
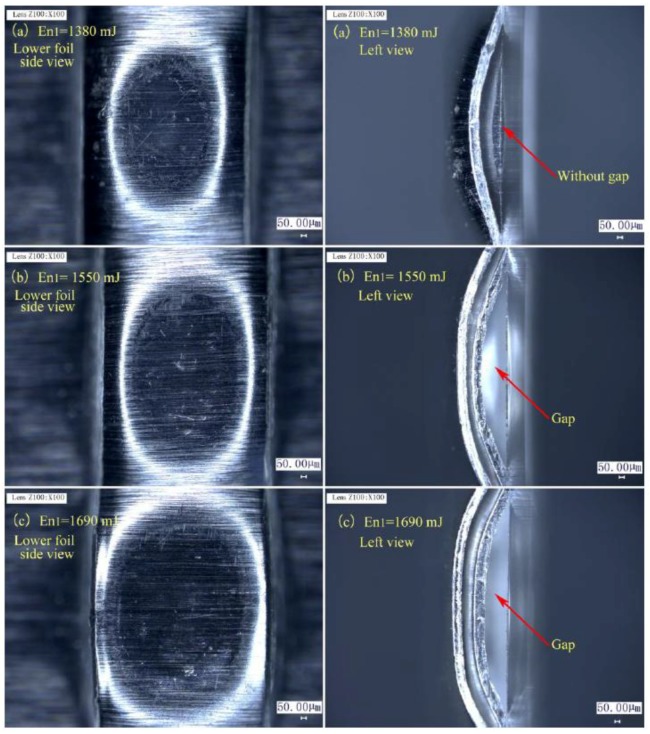
Preliminary joints produced under H of 500 μm and three different En_1_: (**a**) En_1_ = 1380 mJ; (**b**) En_1_ = 1550 mJ; (**c**) En_1_ = 1690 mJ.

Figure 19 shows the matching relationship between En_1_ and total thickness, in which H will be selected from the process window in Figure 17 to reduce the number of experiments. The results show that En_1_ must be increased with the increase in total thickness, because thick material needs greater laser energy to guarantee sufficient drawing, shearing, and flattening actions. Figure 19 was acquired by studying preliminary joints formed in Stage 2. The desired joints part indicates that the preliminary joint can be feasibly formed. Figure 20 shows the preliminary joints of 100/100 μm produced under an H value of 500 μm and En_1_ values of 1380, 1550, and 1690 mJ to illustrate the selection of certain process parameters. These values correspond to the points marked by the red rectangle in Figure 19. The formation of the preliminary joint in the lower foil is enhanced with the increase in laser energy. From the left view of three joints in Figure 20, a gap cannot form under an En_1_ of 1380 mJ. The other two En_1_ values, however, can ensure gap formation. In addition, a certain length of the gap along the sheared edge should be ensured and corresponds to the qualified joining. Otherwise, the length of the “Lock” is too short to guarantee torsional resistance.

**Figure 21:**
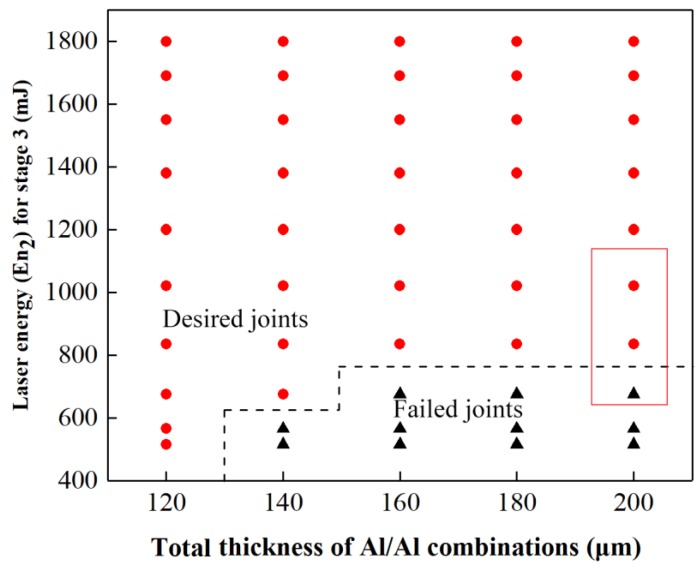
Process window total thickness with En_2_ for Al/Al combinations.

**Figure 22:**
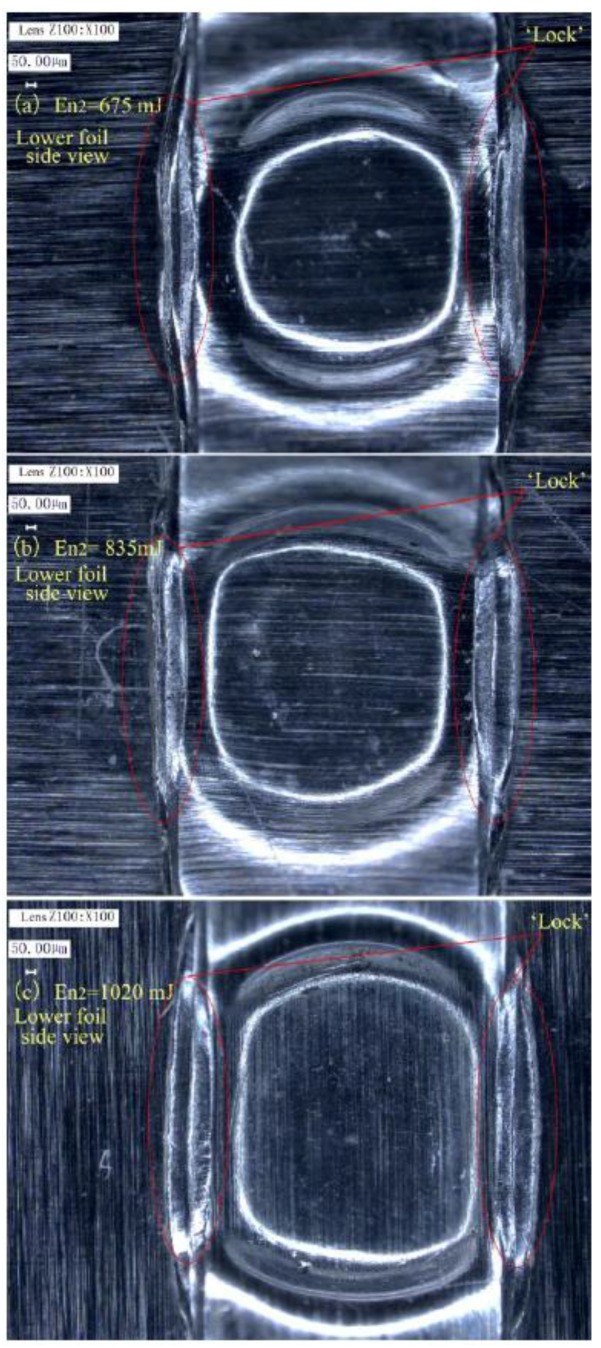
Joints produced under H of 500 μm, En_1_ of 1550 mJ, and three different En_2_: (**a**) En_2_ = 675 mJ; (**b**) En_2_ = 835 mJ; and (**c**) En_2_ = 1020 mJ.

Figure 21 shows the matching relationship between En_2_ and total thickness, in which the tested preliminary joints were all produced by referring to the process windows in Figure 17 and Figure 19. Figure 21 was acquired by investigating the final joints formed in Stage 3. The results show that En_2_ also must be increased with the increase in total thickness to ensure sufficient restriking formation. However, En_2_ is considerably lower than En_1_ because the restriking action of the sheared part has few restrictions. The desired joints part means that a good joint can be successfully formed. The failed joints part indicates that En_2_ cannot ensure a qualified joint. Figure 22 shows the achieved joints of 100/100 μm produced by En_1_ of 1550 mJ, H of 500 μm, and different En_2_ of 675, 835, 1020 Mj. These joints belong to the part marked by red rectangles in Figure 21. The joint produced with an En_2_ of 675 mJ has a smaller formation on the jointing center than the joints produced with the other two En_2_ values. This result indicates that only the energy on the laser spot center enables suitable restriking actions. Moreover, the “Lock” of the joint produced under the En_2_ of 675 mJ did not fit flatly and tightly on the raw foil. These characteristics reflect that the energy at the laser spot edge was insufficient for restriking actions and will damage joining quality. Although the joint produced under 835 mJ was smaller than that produced under 1020 mJ, the joint produced under 835 mJ was a qualified sufficient center formation and tight “Lock”.

Therefore, the qualified preliminary joints must form a sufficiently large gap, and qualified final joints must form a sufficiently long and flat “Lock”. The unqualified joints were all produced through insufficient drawing, shearing, and restriking actions that result from insufficient En_1_, En_2_, and H values. The minimum value of the three influential process parameters must be determined to acquire a qualified joint for certain combinations. This factor can provide a reasonable range of process parameters for subsequent optimization. Additionally, H must first be determined given its fundamental influence on joint formation. The three process parameters all need to be increased as total thickness is increased. Spacer thickness H must be at least twice the total thickness.

#### 3.3.2. Mechanical Properties of Al/Al Joints

The mechanical properties of the micro-shear clinching joints are important because they are used as direct evaluation standards for joint performance and affect process application. The qualified joints can be tested given that the process windows have been acquired. The employed process parameters for different tested Al/Al combinations are listed in Table 9. The selected feasible points in the process window are all close to the boundary line between the desired and failed joint parts. This condition shows that the values of H, En_1_, and En_2_ are all as small as possible. Parallel shear strength and perpendicular shear strength are used to indicate two typical mechanical properties of the joint along a single-lap shearing test direction. The mean value of three samples for parallel shear strength and perpendicular shear strength is noted for every combination.

**Figure 23:**
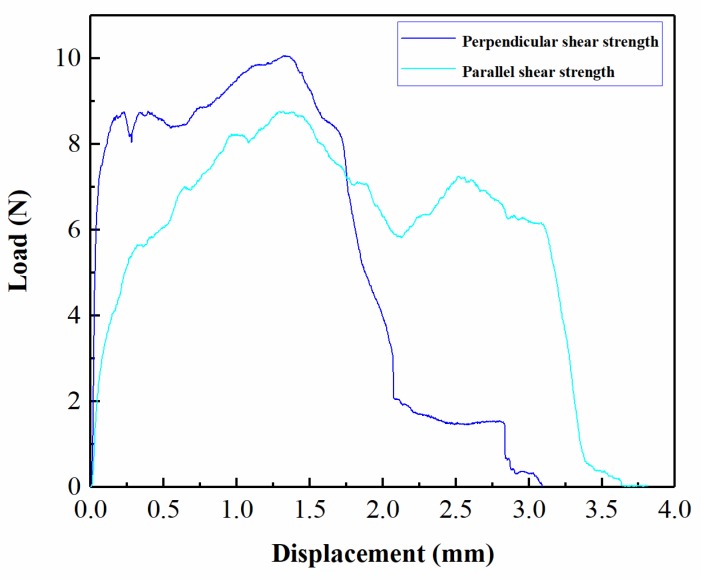
Load–displacement curves for parallel shear strength and perpendicular shear strength for joints with a thickness combination of 100/100 μm (H = 500 μm, En_1_ = 1690 mJ, En_2_ = 1020 mJ).

The load–displacement curves of the two kinds of strengths for joints with a thickness combination of 100/100 μm are shown in Figure 23. The maximum shear strength point of each curve is used to represent joint strength. The loading curve for parallel shear strength first increases slowly as displacement is increased until it reaches the maximum point. This process is the resistance of “Lock”, wherein the wide “Lock” of the upper foil passes the narrow-sheared port of the lower foil. Afterward, the loading curve decreases slowly and then increases to the next-highest peak point. This behavior corresponds to the accumulation of plastic deformation in “Lock” at the narrow port. Finally, the curve decreases to zero. Parallel shear strength has a low value of loading stiffness. The loading curve for perpendicular shear strength first increases rapidly to the second largest peak point, which represents the contact resistance between the sheared part of the upper foil and the raw part of the lower foil. After a period of slow and relatively stable change that results from plastic and shears deformation, the loading curve reaches the maximum point and then decreases to zero. Perpendicular shear strength has a larger loading stiffness than parallel shear strength. This characteristic indicates that the joint is more fastened in the perpendicular direction than in parallel direction. The load–displacement curves of other combinations also exhibit this feature because of similarities across joint shapes.

**Figure 24:**
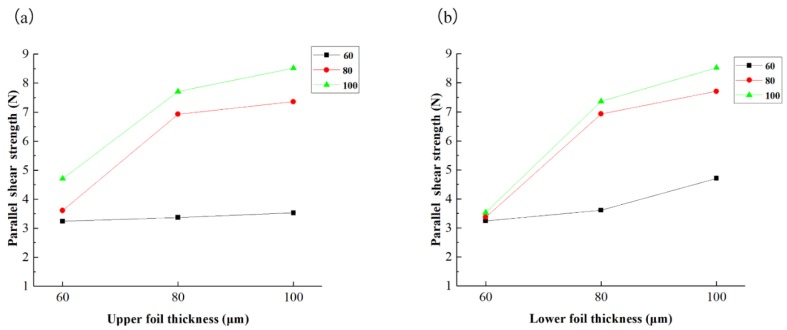
Parallel shear strength of different thickness combinations: (**a**) influence of upper foil thickness and (**b**) influence of lower foil thickness.

The parallel shear strength and perpendicular shear strength for every combination are acquired and are listed in Table 10. This table shows that perpendicular shear strength was always greater than parallel shear strength. The influences of upper and lower foil thicknesses on parallel shear strength are presented in Figure 24a,b, respectively. As shown in Figure 24a, when the lower foil thickness is 60 μm and the upper foil thickness increases from 60 μm to 100 μm, parallel shear strength does not drastically increase because in the parallel shear test, the sheared port of the lower foil provides resistance that easily reaches the upper limit and restrains the maximum point. Strength increases with the increase in upper foil thickness when the lower foil thickness is 80 μm or 100 μm. The resistance between the two foils accordingly increases because the lower foil is sufficiently strong to resist the upper foil. As illustrated in Figure 24b, for every kind of upper foil thickness, strength increases when the lower foil thickness increases from 60 μm to 100 μm. Compared with the lower foil, the upper foil thickness does not limit joint strength. The “Lock” of the upper foil can be strengthened by considerable plastic deformation when a foil with a certain thickness is employed as the upper foil. However, the strength of the sheared port of the lower foil was not improved by shearing actions when the foil was employed as the lower foil. Therefore, parallel shear strength cannot be improved by simply increasing upper foil thickness when the lower foil was weaker than the upper foil. Parallel shear strength can be improved by increasing the thickness of the lower foil when the upper foil is weak.

**Figure 25:**
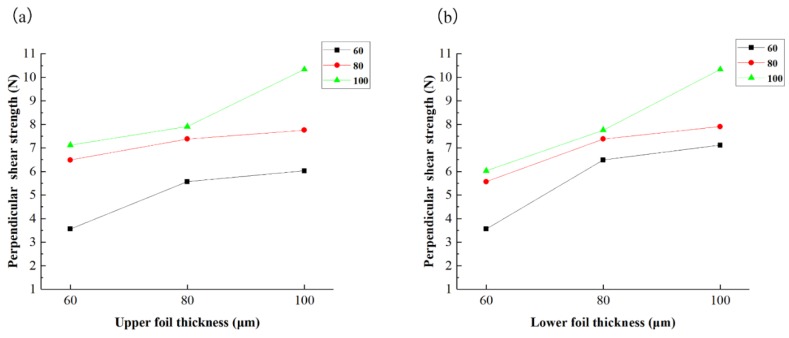
Perpendicular shear strength of different thickness combinations: (**a**) influence of upper foil thickness; (**b**) influence of lower foil thickness.

The influence of upper and lower foil thicknesses on perpendicular shear strength is shown in Figure 25. Strength can be increased by increasing the thickness of the upper foil or lower foil. The weak foil does not impose any resistance limit on different combinations because the resistance area under perpendicular shear is greater than that under parallel shear. This condition reduces the effect exerted by the weak foil. Therefore, perpendicular shear strength can be improved by increasing the thickness of the lower foil or upper foil.

**Figure 26:**
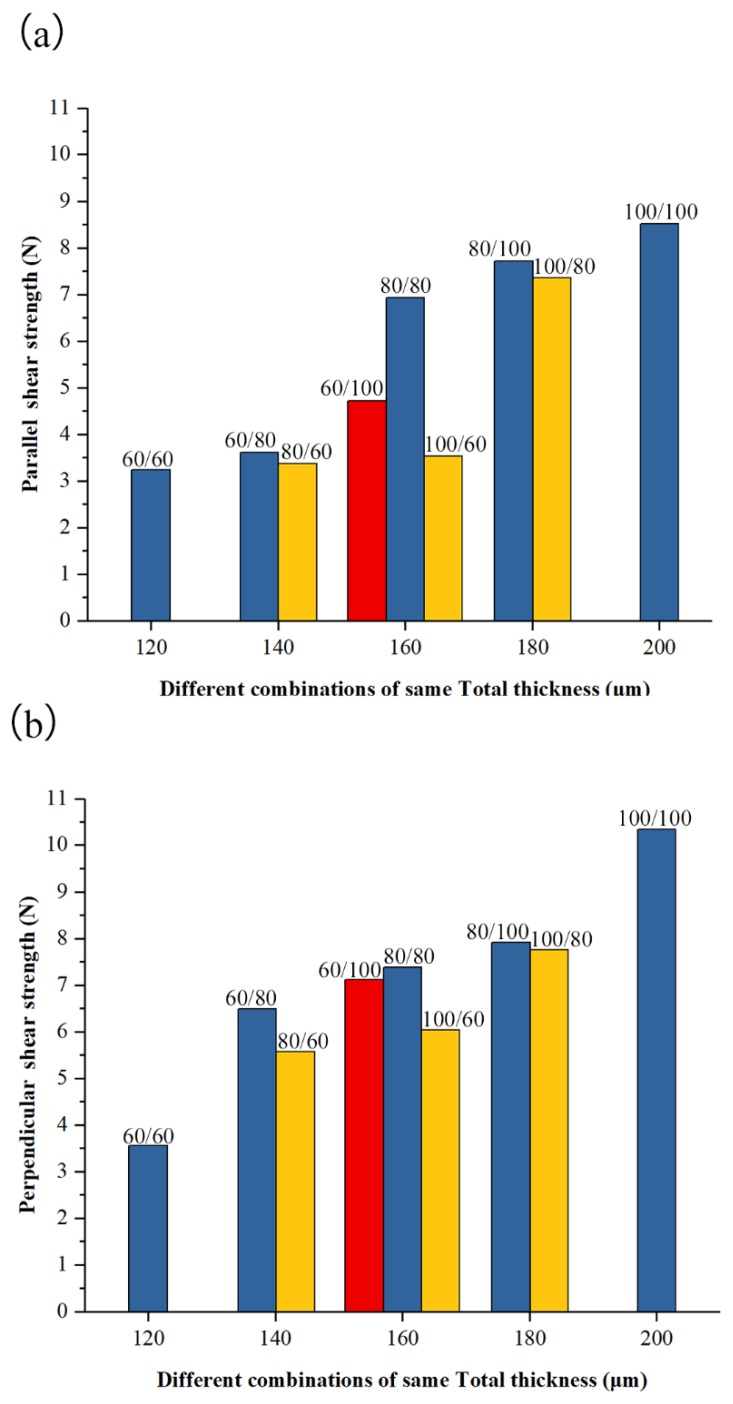
Shear strength of different Al/Al combinations of same total thickness: (**a**) parallel shear strength and (**b**) perpendicular shear strength.

The parallel and perpendicular shear strengths of different combinations with the same Total thicknesses are presented in Figure 26 to illustrate the influence of the thickness combination of the upper and lower foils. The increase in parallel and perpendicular shear strength with the increase in the total thickness of the combination is similar to the increase shown by traditional shear clinching reported by Pedreschi et al. [17]. However, the thickness distribution of the upper and lower foils has a considerable influence. The shear strength of certain combinations of total thicknesses is higher than that of others when the thickness of the upper and lower foils is similar and the foil with higher thickness is used as the lower foil. Therefore, when Total thickness is determined, two foils with similar thickness must be employed, and the foil with higher thickness must be used as the lower foil to obtain high strength.

Therefore, the mechanical properties of the micro-shear clinching joint are related to shear directions and are influenced by the thicknesses of the upper and lower foils.

#### 3.3.3. Failure Modes of Al/Al Joints

Differences between parallel and perpendicular shear strengths cause different failure modes and, thus, are important for the failure analysis of Al/Al joints.

**Figure 27:**
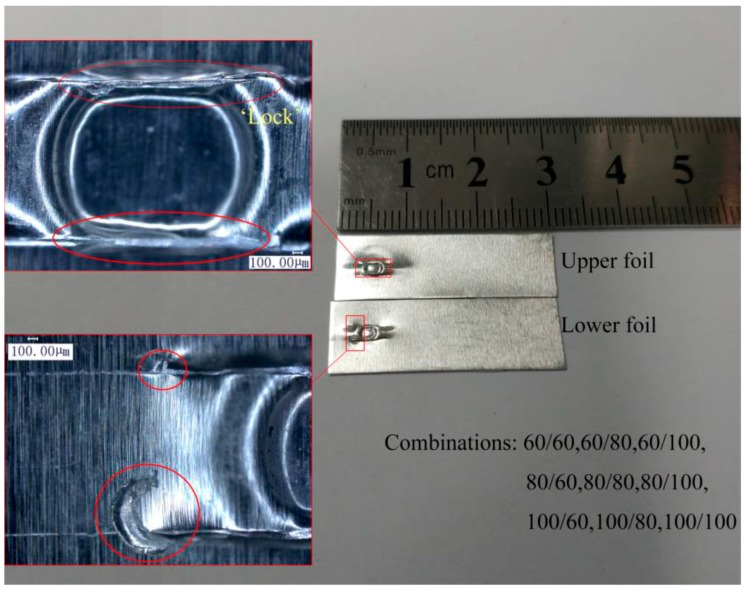
Failure mode of the parallel shear strength test.

Only one failure mode was observed in the parallel shear test, as shown in Figure 27. During the test, the resistance force originated from the widened “Lock” of the upper foil that passes the narrow-sheared port of the lower foil. As shown in Figure 27, the deformed “Lock” of the upper foil is smoothened or sheared by the lower foil, and the residual “Lock” of the upper foil is inlaid on the sheared port of the lower foil.

**Figure 28:**
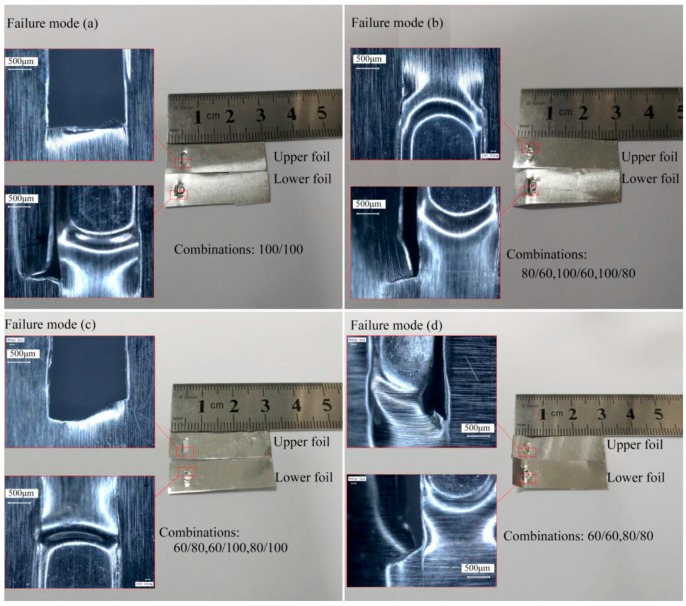
Four kinds of failure modes of perpendicular shear strength test.

As illustrated in Figure 28, four kinds of failure modes are observed in the perpendicular shear test. During the test, the resistance force originates from the sheared part of the upper foil in contact with the raw part of the lower foil. In failure mode (a), the sheared part of the upper foil was completely sheared off, and the lower foil was pulled out of the cracks. Only the 100/100 μm combination undergoes this failure mode. The sheared part of the upper foil was completely sheared by the raw part of the lower foil despite having been strengthened. In failure mode (b), the sheared part of the upper foil appeared intact without noticeable deformation damage. However, the lower foil was pulled out of the cracks and exhibited bending deformation. Combinations with thick upper foils and thin lower foils undergo failure mode (b) because the raw material and work hardness increase the strength of the contact part of upper foil to levels higher than that of the lower foil. In failure mode (c), the sheared part of the upper foil was completely sheared off. However, the lower foil was intact. This mode was presented by combinations with thin upper foils and thick lower foils and contradicts failure mode (b), because although the work hardness of the raw part of the lower foil was less than that of the upper foil, the contact part of the lower foil was stronger than the upper foil as a result of differences in thickness. In failure mode (d), the upper and lower foils were pulled out of the cracks. The 60/60 and 80/80 μm combinations exhibited this mode because two foils with similar thicknesses cause similar resistance deformation. Comparing failure mode (a) with (d) reveals that the upper and lower foils have different failure modes despite having similar thicknesses. This observation shows that the lower foil in failure mode (d) produced significant bending deformation. However, the lower foil in failure mode (a) remained flat. The difference between these two modes may be attributed to a certain degree of dislocation of the shear forces on the two foils that occurs because of the special test sample structure during the shear process. The lower foil with a thickness of 100 μm was sufficiently strong to resist this dislocation force. Lower foils with a thicknesses of less than 100 μm bend because of dislocation force. Bending deformation also occurs in the sheared part of the upper foil undergoing failure mode (d) for the same reason.

Therefore, the failure modes in the parallel shear test for micro-shear clinching joints are only related to the joint structure. The failure modes of the perpendicular shear test were related to joint structure and to the thicknesses and thickness differences of two foils.

### 3.4. Joint Characteristics of Dissimilar Materials

Al foil with a thickness of 60 μm and Cu foil with a thickness of 60 μm can be joined through micro-shear clinching in accordance with the process windows of Al/Al. Thus, the acquired process windows have a high reference value for joining dissimilar materials and can drastically reduce the number of trial experiments.

**Figure 29:**
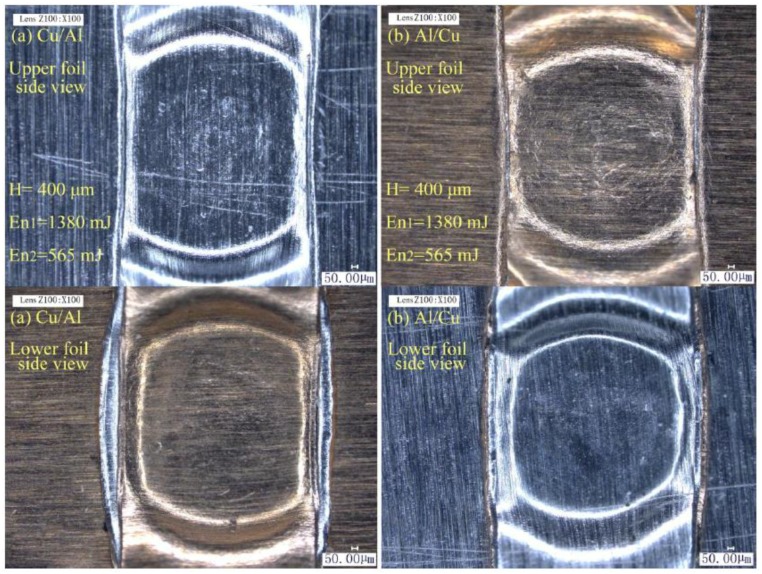
The joints produced by micro-shearing clinching: (**a**) Cu/Al; (**b**) Al/Cu.

Although total thickness was still 120 μm, the strength of the combinations with Cu foils were greater. High H, En_1_, and En_2_ employed to form Al/Cu and Cu/Al joints by micro-shear clinching are shown in Figure 29. These joints have different appearances although they were formed under process parameters. The size and “Lock” of the upper foil formation of the Al/Cu joint were larger than those of the Cu/Al joint. The main drawing actions concentrate on the upper foil, and the lower foil was first sheared with drawing and necking actions because the upper foil was not in direct contact with the shearing mold. Caused by lower plasticity and greater strength of Cu foil than Al foil, Cu foil had less formation than Al foil under the same process parameters. Therefore, under the same process parameters, the upper Al foil can still exhibit sufficient drawing and shearing formation and acquire a large joint size and “Lock” when Cu foil was employed as the lower foil. However, minimal parts of Cu foil undergo sufficient formation when it is employed as the upper foil, thus reducing the joint size and “Lock”. The parallel and perpendicular shear strength of the two kinds of joints are presented in Table 11. The strength of Al/Cu is stronger than that of the Cu/Al, and the strength of Cu/Al is higher than that of Al/Al for 60/60 μm combinations. The rules that influence the joint strength of these two kinds of joints are based on the strength difference between the upper and lower foil and are the same as those that influence the joint strength of Al/Al combinations. The parallel strength of the joint does not drastically increase when the lower foil is Al foil with a thickness of 60 μm because of the upper limit of the sheared port resistance on the lower foil. The perpendicular strengths of the two kinds of joints increased because one of the two foils was Cu foil, which is stronger than the upper or lower foil of the basic Al/Al combination. Therefore, Cu foil with relatively weak plasticity must be set as the lower foil to guarantee the sufficient formation of the upper foil and joint strength when Al and Cu foils have the same thickness. The strength difference of the upper and lower foil in Al/Cu and Cu/Al corresponds to the thickness difference of upper and lower foil in the Al/Al combination. This phenomenon influences joint strength.

**Figure 30:**
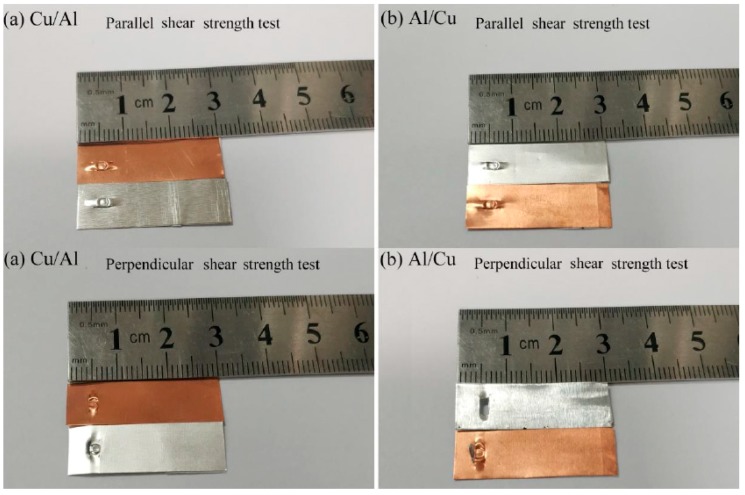
The failure modes of Cu/Al and Al/Cu by parallel and perpendicular shear strength test: (**a**) Cu/Al; (**b**) Al/Cu.

As shown in Figure 30, the parallel shear strength test failure modes of Al/Cu and Cu/Al are the same as those exhibited by Al/Al combinations. The perpendicular shear strength test failure modes of Cu/Al belong to failure mode (b) because the upper foil is stronger than the lower foil. The perpendicular shear strength test failure mode of Al/Cu corresponds to failure mode (c) because the lower foil is stronger than the upper foil.

Therefore, the rules that influence the joint strength and failure modes of the two joints based on Cu and Al foil with same thickness are similar to those that influence Al/Al combinations, wherein the thickness difference of the upper and lower foil in Al/Al combination corresponds to the strength difference of the upper and lower foil in Al/Cu and Cu/Al.

## 4. Conclusions

1. The soft punch thickness of 100 μm was more suitable for micro-shear clinching than other thicknesses. The optimal number of laser pulses on the upper foil side and the lower foil side were two and one, respectively, for ensuring joint deformation and process efficiency.
2. Laser pulse in Stage 1 provides the major contribution of the two foils. From Stage 1 to Stage 3, the thickness of the two foils continuously decreases, but the thickness of two foils do not have much difference in Stage 2 and Stage 3; in the A–A section, from the joint center to the edge, the thickness first decreased and then increased to raw foil thickness, and the thinnest part was located next to the center; in the B–B section, the thinnest part of the upper foil was near the joint edge, but the thickness of the lower foil was relatively uniform. Upper foil bore more tensile stress than the lower foil during the whole process.
3. The micro-shear clinching process windows for Al/Al combinations indicated that three influential parameters—H, En_1_, and En_2_—were all increased as total thickness increases. H needs to be firstly determined, which will vastly restrict the action of En_1_. Spacer thickness H must be at least twice the total thickness.
4. Perpendicular shear strength was always higher and had a larger loading stiffness than parallel shear strength of different Al/Al combinations. When the total thickness is constant, the joint must employ foils with similar thicknesses, and the foil with higher thickness should be used as the lower foil to increase strength.
5. Only one failure mode was observed in the parallel shear test and four failure modes were observed in the perpendicular shear test of the micro-shear clinching of Al/Al combinations. These modes were determined by the thickness of the upper and lower foils and thickness differences of two foils.
6. The difference between the thicknesses of the upper and lower foils in Al/Al combinations with the same thicknesses corresponds to the difference between the strengths of the upper and lower foils in Al/Cu and Cu/Al combinations and determines joint strength and failure modes in micro-shear clinching.

## Figures and Tables

**Table 1:** Main parameters of INNOLAS Nd-YAG Spitlight 2000 laser.

Pulse Energy	Energy Stability	Wave Length	Pulse Width	Spot Diameter
80~1800 mJ	<±1%	1064 nm	8 ns	9 mm

**Table 2:** Constant experimental parameters in micro-shear clinching.

Experimental Parameter	Value
Laser spot diameter (mm)	2
Blank holder pressure (N)	12
Confinement layer thickness (mm)	3
Ablative layer thickness (μm)	10
Width between shearing edges (mm)	1.7
Shearing mold thickness (μm)	200

**Table 3:** Soft punch thickness and other experimental process parameters.

Process Parameter	Value
Soft punch thickness, Ts (μm)	0, 100, 200, 300
Laser energy, En (mJ)	1380
Number of laser pulses (upper foil side)	1
Spacer thickness, H (μm)	300
Upper foil thickness (Al, μm)	60
Lower foil thickness (Al, μm)	60

**Table 4:** Number of laser pulses on the upper foil side and other experimental process parameters.

Process Parameter	Value
Number of laser pulses (upper foil side)	1, 2, 3
Laser energy, En_1_ (mJ)	1380
Spacer thickness, H (μm)	300
Upper foil thickness (Al, μm)	60
Lower foil thickness (Al, μm)	60

**Table 5:** Number of laser pulses on the lower foil side and other experimental process parameters.

Process Parameter	Value
Number of Laser pulses (Lower foil side)	1, 2
Laser energy, En_1_ (mJ)	1380
Laser energy, En_2_ (mJ)	515
Spacer thickness, H (μm)	300
Spacer (Stage 3) thickness (μm)	500
Upper foil thickness (Al, μm)	60
Lower foil thickness (Al, μm)	60

**Table 6:** Determined basic process parameters.

Basic Process Parameters	Value
Soft punch thickness, Ts (μm)	100
Number of laser pulses (upper foil side)	2
Number of laser pulses (lower foil side)	1

**Table 7:** Process parameters.

Process Parameter	Value
Laser energy, En_1_ (mJ)	1380
Laser energy, En_2_ (mJ)	515
Spacer thickness, H (μm)	300
Spacer (Stage 3) thickness (μm)	500
Upper foil thickness (Al, μm)	60
Lower foil thickness (Al, μm)	60

**Table 8:** Experimental process parameters.

Process Parameter	Value
Laser energy, En_1_ (mJ)	515, 565, 675, 835, 1020, 1200, 1380, 1550, 1690, 1800
Laser energy, En_2_ (mJ)	515, 565, 675, 835, 1020, 1200, 1380, 1550, 1690, 1800
Spacer thickness, H (μm)	200, 300, 400, 500
Spacer (Stage 3) thickness (μm)	400, 500, 600, 700
Upper foil thickness (Al, μm)	60, 80, 100
Lower foil thickness (Al, μm)	60, 80, 100
Total thickness (μm)	120, 140, 160, 180, 200

**Table 9:** Process parameters for different tested Al/Al combinations.

Al/Al (μm)	H (μm)	En_1_ (mJ)	En_2_ (mJ)	Spacer (Stage 3) Thickness (μm)
60/60	300	1380	515	500
60/80	400	1380	675	600
60/100	400	1550	835	600
80/60	400	1380	675	600
80/80	400	1550	835	600
80/100	400	1690	835	600
100/60	400	1550	835	600
100/80	400	1690	835	600
100/100	500	1550	835	700

**Table 10:** The parallel and perpendicular shear strength of tested Al/Al combinations.

Al/Al Combinations (μm)	Parallel Shear Strength (N)	Perpendicular Shear Strength (N)
60/60	3.24	3.56
60/80	3.61	6.49
60/100	4.71	7.12
80/60	3.37	5.57
80/80	6.93	7.38
80/100	7.71	7.91
100/60	3.53	6.03
100/80	7.36	7.76
100/100	8.52	10.34

**Table 11:** The parallel and perpendicular shear strength of Al/Cu and Cu/Al.

	Al/Cu	Cu/Al
Parallel shear strength (N)	3.89	3.34
Perpendicular shear strength (N)	4.55	3.78
